# Suspensions of antibiotics in self-emulsifying oils as a novel approach to formulate eye drops with substances which undergo hydrolysis in aqueous environment

**DOI:** 10.1080/10717544.2024.2372279

**Published:** 2024-07-11

**Authors:** Katarzyna Krzeminska, Malgorzata Sznitowska, Magdalena Wroblewska, Eliza Wolska, Katarzyna Winnicka

**Affiliations:** a Department of Pharmaceutical Technology, Faculty of Pharmacy, Medical University of Gdansk, Gdansk, Poland; b Department of Pharmaceutical Technology, Faculty of Pharmacy, Medical University of Bialystok, Bialystok, Poland

**Keywords:** Self-emulsifying oils (SEO), cefuroxime sodium, vancomycin hydrochloride, stability, microbiological assay

## Abstract

The aim of this study was to develop eye-drops with cefuroxime (CEF) sodium or vancomycin (VAN) hydrochloride, antibiotics that are instable in water. Anhydrous self-emulsifying oils (SEO) are proposed as a carrier and antibiotics are suspended. In the contact with tear fluid, the formulation should transform into emulsion, with fast dissolution of an antibiotic. CEF or VAN (5% w/w) was suspended in SEO carriers prepared by dissolving surfactants (Tween 20 or Span 80 5% w/w) in Miglyol, castor oil, or olive oil. Formulations with or without sodium citrate (2% w/w) were compared. Six-months or 1-year stability tests were carried out at 40 °C. The content of CEF and VAN was evaluated using HPLC and the potency of the antibiotic was assessed with agar diffusion method. In contact with water, drug particles suspended in SEO dissolved rapidly and o/w emulsion was formed. After 1-year at 40 °C, the content of degradation products was at most 0.5% in CEF and 4.0% in VAN formulations. The agar diffusion assay has shown that CEF and VAN loaded into SEO retained its potency against the sensitive microorganisms comparable to an aqueous solution. Therefore, SEO can be used as a novel carrier for the active substances which may not require improved solubility or absorption but need to be protected from moisture. This is a formulation that can be produced on industrial scale, with no limitation of stability or drug concentration.

## 1. Introduction

Self-emulsifying oils (SEO) also called self-emulsifying drug delivery systems (SEDDS) are water-free isotropic mixtures of oils, surfactants and occasionally co-surfactants. Following dilution with the aqueous media, a fine oil-in-water (o/w) emulsion is created, which can also be obtained *in vivo* at the site of administration (Salawi, 2022). Safety of SEO for ocular administration has already been confirmed (Czajkowska-Kośnik et al., 2015; Rasoanirina et al., 2020; Wolska et al., 2023). Until now, such systems have been studied as carriers for the drug substances poorly soluble in water such as prednisolone (Tiwari et al., 2019), indomethacin, acyclovir, hydrocortisone (Czajkowska-Kośnik & Sznitowska, 2009) or econazole (Elbahwy et al., 2018), when dissolution rate-limited absorption is a case. However, the anhydrous nature of SEO provides the environment that might reduce significant degradation of water-sensitive drugs, regardless if they are soluble or insoluble in water.

Among water-sensitive drugs are many antibiotics that decompose by hydrolysis, what leads to loss of their therapeutic potential. Antibiotics that are hydrolyzable in aqueous media include: cephalosporins, penicillins, carbapenems, tetracyclines, or peptides (Samara et al., 2017) what means that they cannot be used as commercially available aqueous solutions (or emulsions) and the final liquid formulation must be prepared ex tempore from powder or granules.

However, this approach, although applied for injections or pediatric oral forms, is not applicable for eye drops since mixing a powder with the solvent requires aseptic technique, which cannot be ensured outside a medical setting. Therefore, the objective of the current project is focusing on the development of SEO-based, liquid ready-to-use formulations for antibiotics or other drugs which require moisture protection during storage. For the purpose of this study, cefuroxime (CEF) sodium and vancomycin (VAN) hydrochloride were chosen as model water-sensitive drugs.

Cefuroxime is used as a sodium salt. It is a cephalosporin antibiotic, from a subgroup within the broader category of beta-lactam antibiotics, with the activity against staphylococci and Gram-negative bacteria (Marshall & Blair, 1999). Since 2012, when the first formulation as anterior chamber injections was registered in Europe (Aprokam^®^, powder for solution for injection, *Thea*), the availability of this drug form has significantly spread its use in ophthalmology (Rękas et al., 2022).

In hospital pharmacies, CEF eye drops are prepared from sterile powder for solution for injection, typically at a concentration of 1% w/w. Also 5% fortified solutions to treat suppurative keratitis are prepared by dissolving 500 mg of lyophilized antibiotic in 2.5 ml of sterile water. Following this, 7.5 ml of non-preserved sterile artificial tears are added (Kodym et al., 2011).

**Figure 1.**
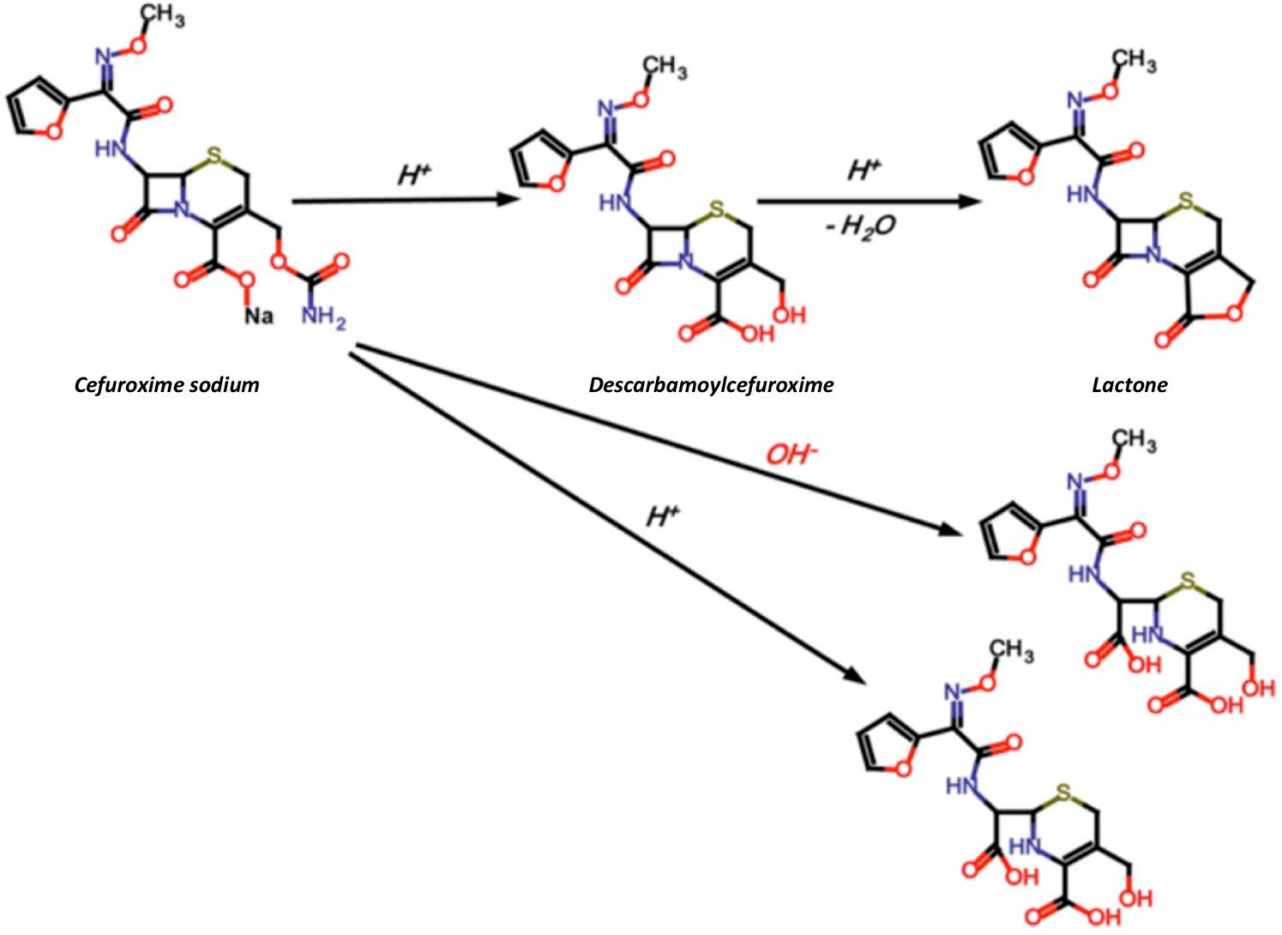
Degradation pathway of cefuroxime sodium (Wang & Notari, 1994).

Figure 1 shows the decomposition scheme of CEF (Sharaf El-Din et al., 2021). The mechanism of CEF degradation is dependent on the pH of the solution (the best stability is at pH 4.5–7.3), temperature, light and type of packaging (Wang & Notari, 1994; Kodym et al., 2011). Degradation of cephalosporins involves dissociation of the lactam ring when the pH is no more than 3.5 (Yamana & Tsuji, 1976). At higher pH values, dissociation of the carbamate group at the C3 position occurs (Wozniak & Hicks, 1991). The main degradation product of CEF is descarbamoyl cefuroxime, which exhibits antimicrobial activity due to preserved beta-lactam ring. It was previously reported that the shelf life of CEF eye drops obtained from powder for solution for injection was: in the freezer (about −18 °C) at least 12 months, in the refrigerator (−2 °C) 4 weeks, and at room temperature only 24 hours (Oldham, 1991).

Vancomycin is a glycopeptide, an antibiotic containing sugar molecules in their structure. Its use in ophthalmology is restrained to most severe bacterial infections caused by Gram-positive organisms (Karampatakis et al., 2009; Montes et al., 2016). As the antibiotics resistance worldwide is still rising, it is exceptionally useful in patients with external ocular infections unresponsive to the treatment of fluoroquinolone eye drops (Sotozono et al., 2013; Yousry et al., 2016). Vancomycin, as a hydrochloride salt, is typically used in concentration from 1 to 5%.

**Figure 2.**
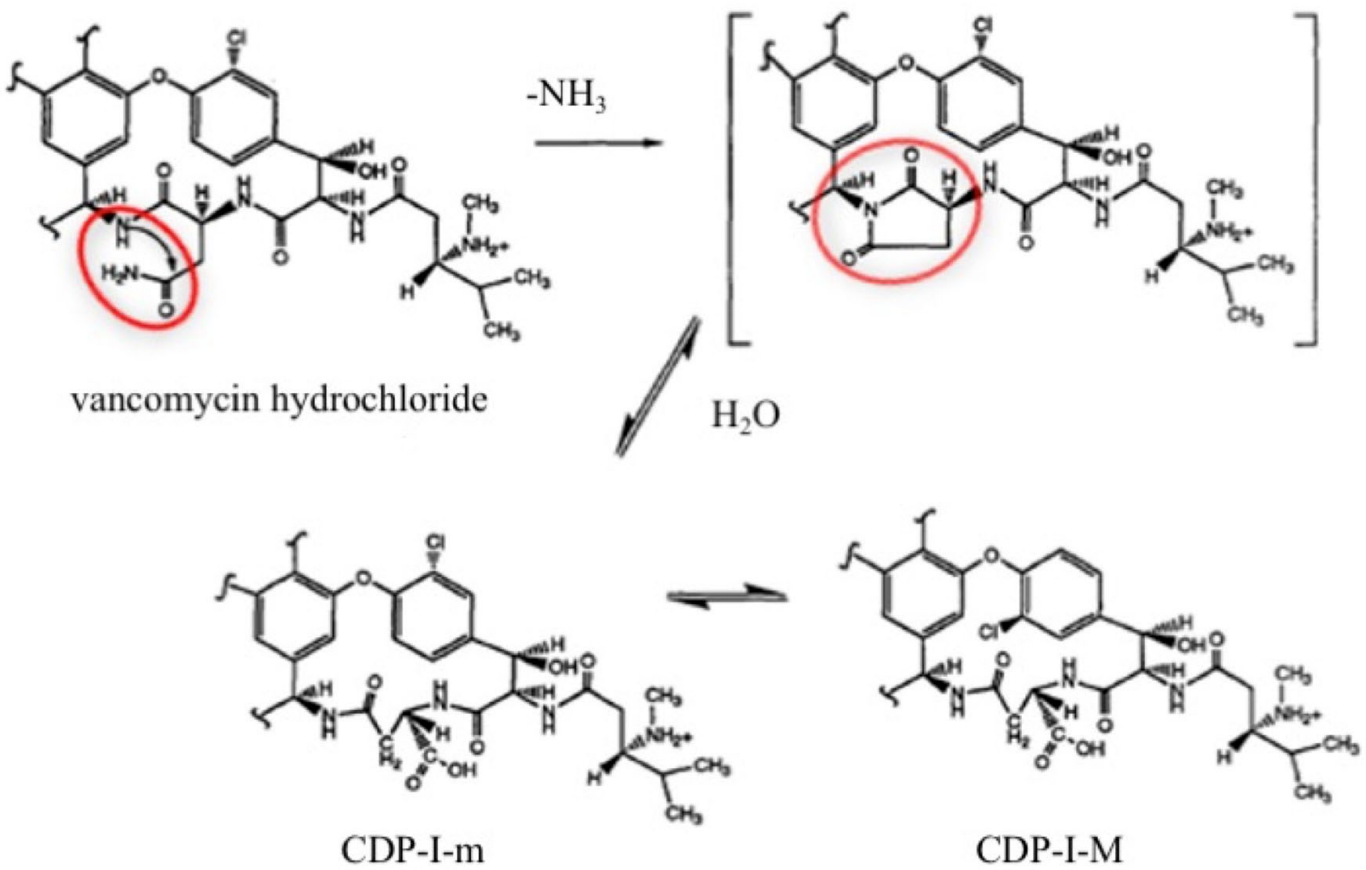
Mechanism of VAN degradation in aqueous environment (Antipas et al., 1994).

VAN is instable in water and the compounded eye-drops should be frozen for storage longer than 4 weeks (Fuhrman & Stroman, 1998; Lin et al., 2005; Chédru-Legros et al., 2007). A schematic degradation of VAN in water is presented in Figure 2. VAN is degraded to CDP-I (crystalline degradation product), which exists as two isomers CDP-I-M (major crystalline degradation product) and CDP-I-m (minor crystalline degradation product), which differ in the positioning of the chlorine atom in space. The degradation products of VAN are devoid of antimicrobial activity on account of the hydrolysis, deamidation, and rearrangement of the asparagine moiety in the side chain of VAN (Klapkova et al., 2020; Jakaria et al., 2022).

Both antibiotics, VAN and CEF are used in the form of their salts (hydrochloride and sodium, respectively) and are freely soluble in water and insoluble in oil, so the concept assumes that in SEO they are suspended. Only when the formulation comes into contact with the lacrimal fluid, a fine emulsion occurs with spontaneous dissolution of antibiotic. The suspension offers the possibility of increased concentrations of a drug, without being limited by its solubility.

In this study, the chemical stability of the CEF and VAN as the model moisture-labile drugs loaded into SEO carriers was tested and the content assay was performed to determine the potential drug decomposition over time. The antimicrobial potency of antibiotics loaded into SEO carriers was also assessed with an agar diffusion method. Additionally, the problem of homogeneity of the suspension upon storage has been addressed and modification of the formulations with a deflocculating agent was considered.

## 2. Materials and methods

### 2.1. Materials

Cefuroxime sodium and VAN hydrochloride in the form of powder for injection were purchased from MIP-Pharma (Chephasaar, St. Ingbert, Germany). Polyoxyethylene sorbitan monolaurate (Tween 20) was purchased from Sigma-Aldrich (Steinheim, Germany), sorbitan monooleate (Span 80) from Fluka (Buchs, Switzerland), fractionated coconut oil (Miglyol 812) from Caesar & Loretz (Hilden, Germany), castor oil from Fagron (Kraków, Poland) and olive oil (Davalia oil from Aria Worldwide, Barcelona, Spain). Sodium citrate was obtained from Stanlab (Lublin, Poland).

All chemicals and solvents used for chromatographic analysis were of an analytical grade and were purchased from Avantor (Gliwice, Poland) or Sigma-Aldrich (Steinheim, Germany). Purified water was produced in house with Elix 3 Millipore system (Bedford, MA).

### 2.2. Preparation of tested SEO formulations

Composition of the prepared SEO with CEF and VAN (5% w/w) is presented in Table 1.

**Table 1.** The composition of investigated SEO formulations.

Formulation	Antibiotic		Surfactant		Oil			Additives
	CEF	VAN	Tween 20	Span 80	Miglyol	Olive oil	Castor oil	Sodium citrate
CEF_TwM	5.0		5.0		90.0			
CEF_TwO	5.0		5.0			90.0		
CEF_TwR	5.0		5.0				90.0	
CEF_SpM	5.0			5.0	90.0			
CEF_SpO	5.0			5.0		90.0		
CEF_SpR	5.0			5.0			90.0	
VAN_TwM		5.0	5.0		90.0			
VAN_TwO		5.0	5.0			90.0		
VAN_TwR		5.0	5.0				90.0	
VAN_SpM		5.0		5.0	90.0			
VAN_SpO		5.0		5.0		90.0		
VAN_SpR		5.0		5.0			90.0	
CEF_TwMc	5.0		5.0		88.0			2.0
VAN_TwMc		5.0	5.0		88.0			2.0

The suspensions were developed aseptically using sterile ingredients. The SEO carriers were prepared by mixing oil (olive oil, Miglyol, and castor oil) with surfactant (Span 80 or Tween 20 at concentration of 5% w/w) using a magnetic stirrer (30 minutes, 50 °C). Powders of CEF, VAN, and sodium citrate were micronized by ball milling with zirconium balls in a plastic container (Planetary Mixer ARE-250, Thinky, Tokyo, Japan) in two cycles (2000 rpm, two minutes each). Then, SEO carrier was added to the container with the CEF or VAN (with or without addition of sodium citrate) and mixed in two cycles, each lasting two minutes at 2000 rpm. When the suspension was deemed homogeneous, the zirconium balls were separated from the formulation using a sieve with a 80 μm mesh size. In the final step, the formulations were dispensed in 10 g portions into glass vials and closed tightly.

### 2.3. Storage of SEO suspensions and stability test

The prepared formulations were stored for 1 year (SEO formulations without sodium citrate) or 6 months (CEF_TwMc and VAN_TwMc) in a climate chamber at a temperature of 25 °C (relative humidity RH = 60%) and 40 °C (RH 75%) (climatic chamber KBF 115 Binder, Tuttlingen, Germany). The suspensions were prepared in triplicate for each storage condition.

Right after preparation (T0) and again after 1, 3, and 6 months and 1 year of storage, the water was added to the SEO formulations to obtain emulsions which were assessed visually, chromatographically, and physicochemically (pH, zeta potential, and median droplet size).

### 2.4. Physicochemical studies of SEO formulations

#### 2.4.1. Visual observation

The self-emulsification performance upon dilution of VAN and CEF SEO suspensions with water was evaluated visually for the appearance of signs of breaking the emulsion. Samples were mixed with water (1:10 or 1:100 w/w) on a vortex for 30 seconds, and the time needed for emulsion phase separation was measured.

#### 2.4.2. Microscopic evaluation

Microscopic images of antibiotics powders, SEO suspensions, and water-diluted SEO emulsions (1:10 w/w) were obtained using a fluorescent microscope (Nikon Eclipse i50, Tokyo, Japan). The observations were carried out using Zeiss objectives (Oberkochen, Germany): a ×10 objective for a general overview and a ×40 magnification lens to capture images within a depth range of 25 μm. The size of CEF and VAN particles in both powder and SEO suspensions was measured and calculated using NIS Elements Advanced Research 3.20 software (Nikon Instruments, Tokyo, Japan).

#### 2.4.3. Droplet size measurement

The *d*_50_ parameter (the size at which the cumulative droplet size distribution curve reaches 50% of the dispersed phase volume) was assessed using the laser diffraction technique in a Beckman-Coulter LS 13 320 instrument (Indianapolis, IN) equipped with the polarization intensity differential scattering (PIDS) function. The CEF_SEO and VAN_SEO suspensions were diluted with water in a 1:10 (w/w) ratio. Each measurement was repeated three times.

#### 2.4.4. Zeta potential

The zeta potential was calculated based on electrophoretic mobility and measured using the Zetasizer Nano ZS instrument (Malvern Instruments, Worcestershire, UK) at 25 °C, following the dilution of SEO in water (1:1000 w/w). The obtained result was the average of three repetitions of each measurement.

#### 2.4.5. pH

The pH measurements were performed with a pH meter Five Easy F20 (Mettler Toledo, Columbus, OH) calibrated with standard solutions pH of 4.0 and 7.0. The pH of the SEO emulsions (dilution 1:10 w/w) was determined by placing the electrode directly into the sample.

### 2.5. HPLC analysis

The CEF and VAN assay in the prepared formulations was carried out using high-performance liquid chromatography (HPLC) system (Prominence-i LC-2030C 3D, Shimadzu Corporation, Kyoto, Japan) equipped with a UV–vis detector. The HPLC analysis for CEF was conducted according to the European Pharmacopoeia (Ph. Eur. 11.2) with minor modifications concerning the mobile phase composition (Table 2), whereas for VAN, the parameters were determined experimentally based on literature (Jesús Valle et al., 2008).

**Table 2.** HPLC analysis parameters for CEF and VAN.

Parameter	CEF	VAN
Mobile phase	Acetate buffer pH 3.4:acetonitrile (95:5 v/v)	Phosphate buffer pH 3.0:acetonitrile (92:8 v/v)
Detection	Spectrophotometer: 273 nm	Spectrophotometer: 210 nm
Injection	20 μl	20 μl
Flow rate	1.5 ml/min	1.0 ml/min
Retention time	Approximately 10 min	Approximately 8 min
Column + guard column	LiChrospher^®^ 100, C18 (5 µm) 125 × 4 mm	

The content assay was performed to determine drug degradation rate over time. After removal from the climate chamber, the investigated SEO formulations were mixed on a vortex mixer for 30 seconds to obtain a homogeneous SEO suspension. The first step in preparing samples for HPLC involved creating an SEO emulsion by 10-fold (1:10 w/w) dilution. For this purpose, 1.0 g of the emulsion was weighed, diluted with water in a measuring flask to a total volume of 10 ml and again mixed on a vortex mixer for 30 seconds. Then, in three repetitions, 1.0 ml of the obtained dilution was withdrawn to the flask and filled with water up to 50.0 ml. In the final step, 1.0 ml of the 1:500 emulsion was withdrawn, diluted with the mobile phase in a 10 ml volumetric flask and immediately analyzed using HPLC.

For comparison, the stability of CEF and VAN in water (5% w/w solutions) was also tested. The solutions were prepared by dissolving 0.5 g of antibiotic (CEF or VAN) in 9.5 g of water. Then tightly sealed glass vials with solutions were stored at temperatures of 4 °C, 25 °C (RH = 60%) and 40 °C (RH = 75%) and the contents of CEF and VAN were evaluated after 24 h, 7 days, 14 days, 21 days, and 1 month of storage. The final concentration of antibiotic in the analyzed samples was 10 µg/ml.

### 2.6. Microbial assay

The microbiological assay was performed in accordance with the diffusion assay described by European Pharmacopeia with slight modifications. Thirty Petri dishes (diameter 9 cm, height 1 cm) containing inoculated media: 30 ml Mueller Hinton 2 LAB-AGAR™ (Table 3) and 100 µl spore suspension strain (Selectrol^®^, TCS Biosciences, Botolph Claydon, UK) appropriate for the investigated antibiotics were used for testing (Table 4). The inoculum was prepared by suspending several colonies from a fresh 24-hour culture of the strain in a sterile 0.9% NaCl solution to achieve a suspension with a density of 0.5 McFarland (MF) scale, which is approximately 1.5 × 10^8^ cells/ml. The 5 mm diameter holes in the solidified media were cut out using a sterile cork borer to introduce tested emulsions or solutions. On a single Petri dish, there was always one hole applied.

**Table 3.** Composition of Mueller Hinton 2 LAB-AGAR™ (BioMaxima, Lublin, Poland).

Ingredient	Amount (g/l)
Beef extract powder	2.0
Starch	1.5
Casein acid hydrolysate	17.5
Agar	17.0
Water	Up to 1000 ml

**Table 4.** Formulations and microorganisms tested in the microbiological study.

Antibiotic 3.75% (w/w)	Formulation (solution or emulsion)	Storage of SEO	Microorganism
CEF	Solution	T0	*Staphylococcus aureus* ATCC 6538P
	CEF_TwM	T0 and 12 months at 25 °C	
VAN	Solution	T0	*Bacillus subtilis* ATCC 6633
	VAN_TwM	T0 and 12 months at 25 °C	

The 5% SEO suspensions, containing Miglyol and Tween were selected for the study. To confirm their stability, the suspensions stored for 1 year at 40 °C were also analyzed (Table 4). The suspensions were initially diluted with sterile water (3:1) to obtain emulsion and reach the antibiotic concentration of 3.75%. CEF or VAN aqueous solutions of the same concentration, prepared directly before the study, were used as a reference. One hundred microliters of the obtained emulsion or reference solution was placed in the hole of inoculated culture medium, to fill it completely. The number of replications for each studied sample was five. The Petri dishes were incubated at 37 °C for 24 h. The diameters of the circular inhibition zones were measured with a precision of 0.1 mm.

### 2.7. Statistical assessment

The data were analyzed using analysis of variance (ANOVA) for conducting multiple comparisons of the means. The computations were carried out using Statistica 12 (StatSoft, Tulsa, OK). Statistically significant differences were determined for a *p* value of <.05.

## 3. Results and discussion

### 3.1. Characteristics of SEO suspensions

#### 3.1.1. Visual and microscopic analysis

**Figure 3.**
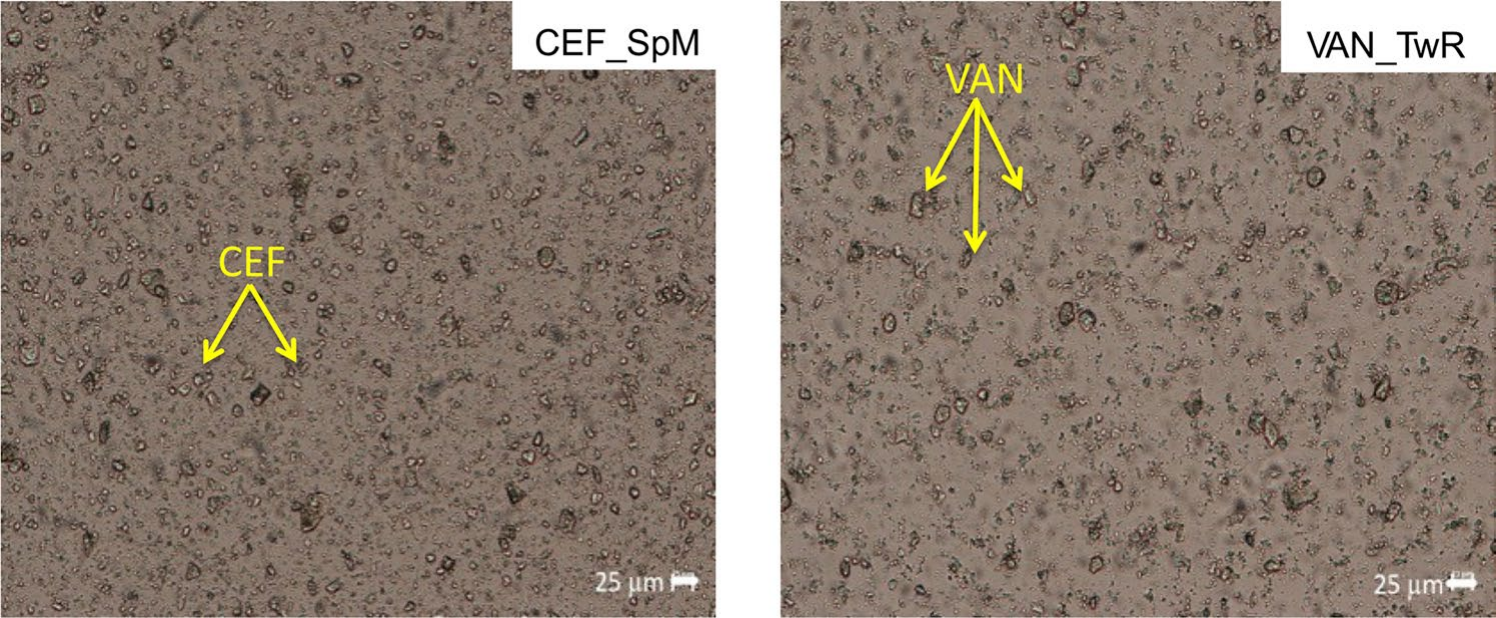
Microscopic images of SEO suspensions with CEF (in SEO consisted of span and Miglyol) and VAN (in SEO consisted of tween and castor oil).

All suspensions as assessed visually, exhibited a homogenous appearance after 30 second manual shaking. Microscopic examination also confirmed a uniform dispersion of CEF and VAN powder in all prepared SEO suspensions (Figure 3). None of the suspended particles of CEF and VAN was larger than 25 µm, with majority smaller than 10 µm.

### 3.2. Characteristics of emulsions obtained by dilution of SEO suspensions

#### 3.2.1. Visual observations

**Figure 4.**
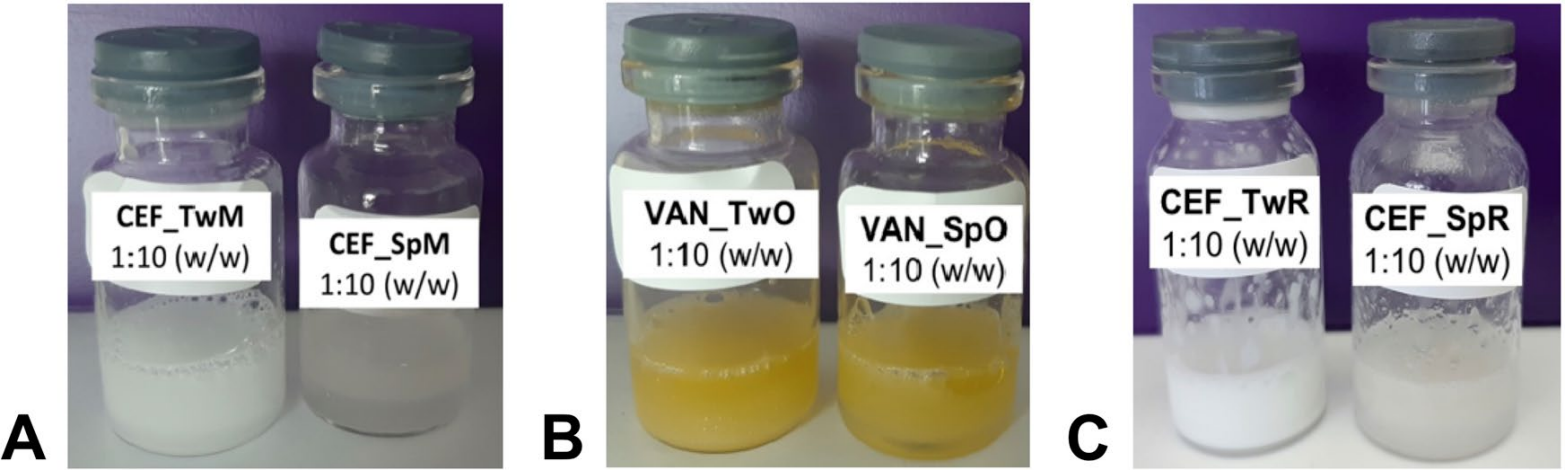
The appearance of the obtained emulsions after dilution of SEO suspensions with water (1:10 w/w). (A) CEF emulsions with Miglyol, (B) VAN emulsions with olive oil, and (C) CEF emulsions with castor oil.

Upon dilution of the suspensions with water, the drugs dissolved immediately, regardless of the dilution ratio. Figure 4 depicts the appearance of the obtained emulsions. The appearance of the emulsions was solely dependent on the composition of the SEO and not on the type of antibiotic.

The Miglyol-based SEO suspensions easily formed milky, white, homogeneous emulsions, regardless of the type of surfactant used (Figure 4(a)). SEO with olive oil produced yellow, homogeneous emulsions (Figure 4(b)). The emulsification of both SEO formulations through vortex mixing was easy. Also emulsions with castor oil and Tween 20 had a homogeneous, milky appearance and exhibited good emulsifying capabilities (Figure 4(c)). However, emulsions with castor oil and Span 80 were challenging to form, despite repeated vortex mixing. The formulations were not homogeneous and large oil droplets were visible to the naked eye on the surface of the emulsion or on the vial walls.

#### 3.2.2. Size of the droplets

**Figure 5.**
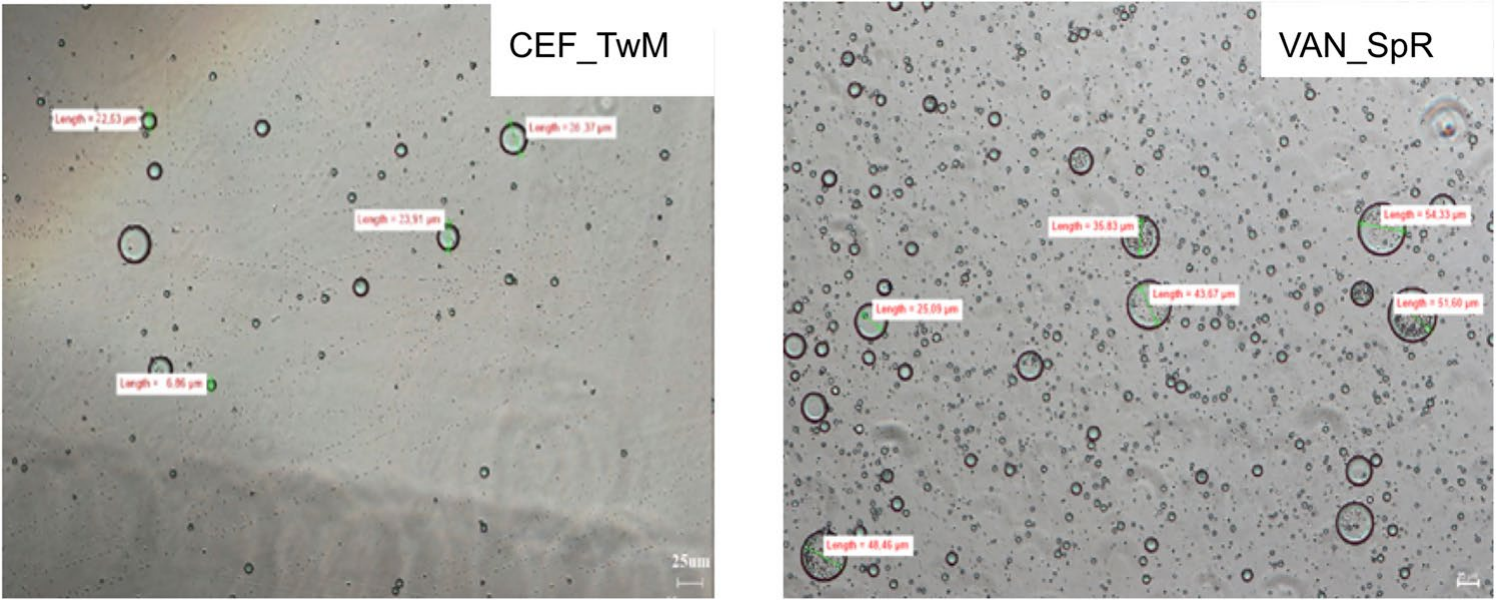
Microscopic images of the emulsions obtained after dilution CEF_TwM (CEF in SEO consisted of tween and Miglyol) and VAN_SpR (VAN in SEO composed of span and castor oil) suspensions with water (1:10 w/w).

As determined in previous studies, regardless of the type of surfactant, the addition of water results in an o/w type of emulsion (Krzemińska & Sznitowska, 2023). In the microscopic images of the obtained emulsions, no significant differences were observed, regardless of their composition and the type of antibiotic they contained (Figure 5). The size of oily droplets was not larger than 30 µm. An exception, as in visual observations, was the castor oil emulsion with Span 80, which exhibited a non-uniform appearance and droplet sizes up to 50 µm.

The results of the measurements performed by a laser diffractometry are shown in Table 5. The largest emulsion droplets (*d*_50_ around 15 µm) were found in the castor oil and Span emulsions (CEF_SpR and VAN_SpR). In the other emulsions, the measured *d*_50_ was less than 10 µm.

**Table 5.** The physicochemical properties of the emulsions obtained after dilution with water (1:10) of VAN and CEF suspensions in SEO.

SEO	Oil	Surfactant	Zeta potential (mV)	pH	*d*_50_ (µm)
*CEF*					
CEF_TwR	Castor oil	Tween 20	−43.4 ± 1.2	6.4	6.5 ± 0.2
CEF_SpR		Span 80	−66.3 ± 1.5	6.4	13.5 ± 1.0
CEF_TwM	Miglyol	Tween 20	−41.7 ± 0.7	6.4	6.2 ± 0.3
CEF_SpM		Span 80	−68.4 ± 1.0	6.4	7.9 ± 0.6
CEF_TwO	Olive oil	Tween 20	−51.0 ± 0.8	6.8	7.8 ± 0.5
CEF_SpO		Span 80	−55.6 ± 1.1	6.5	7.4 ± 0.6
*VAN*					
VAN_TwR	Castor oil	Tween 20	−36.8 ± 0.5	5.4	4.1 ± 0.2
VAN_SpR		Span 80	−47.0 ± 0.7	5.7	14.1 ± 1.0
VAN_TwM	Miglyol	Tween 20	−31.9 ± 0.6	5.7	5.7 ± 0.3
VAN_SpM		Span 80	−40.7 ± 0.7	5.8	4.1 ± 0.4
VAN_TwO	Olive oil	Tween 20	−44.1 ± 0.9	5.6	4.0 ± 0.5
VAN_SpO		Span 80	−49.5 ± 1.1	5.6	4.6 ± 0.5

#### 3.2.3. pH

The pH of the analyzed formulations varied depending on type of antibiotic salt (Table 5): the CEF emulsions had a pH value of approximately 6.4, while in the VAN formulations, the pH was lower, around 5.6.

#### 3.2.4. Zeta potential

The zeta potential of the emulsions resulting from SEO suspensions with CEF significantly varied depending on the composition of the formulations, ranging from −41.7 mV to −68.4 mV. In contrast, in the formulations with VAN, it ranged from −31.9 mV to −49.5 mV (Table 5). In both VAN and CEF emulsions with Span 80, the zeta potential was more negative compared to those with Tween 20, what is in contrast to their faster coalescence and phase separation.

### 3.3. Stability studies

#### 3.3.1. Aqueous solutions

In order to compare the stability of antibiotics in an aqueous solution and SEO suspensions, 5% antibiotic solutions in water were stored for 1 month at both 25 °C and 40 °C.

**Figure 6.**
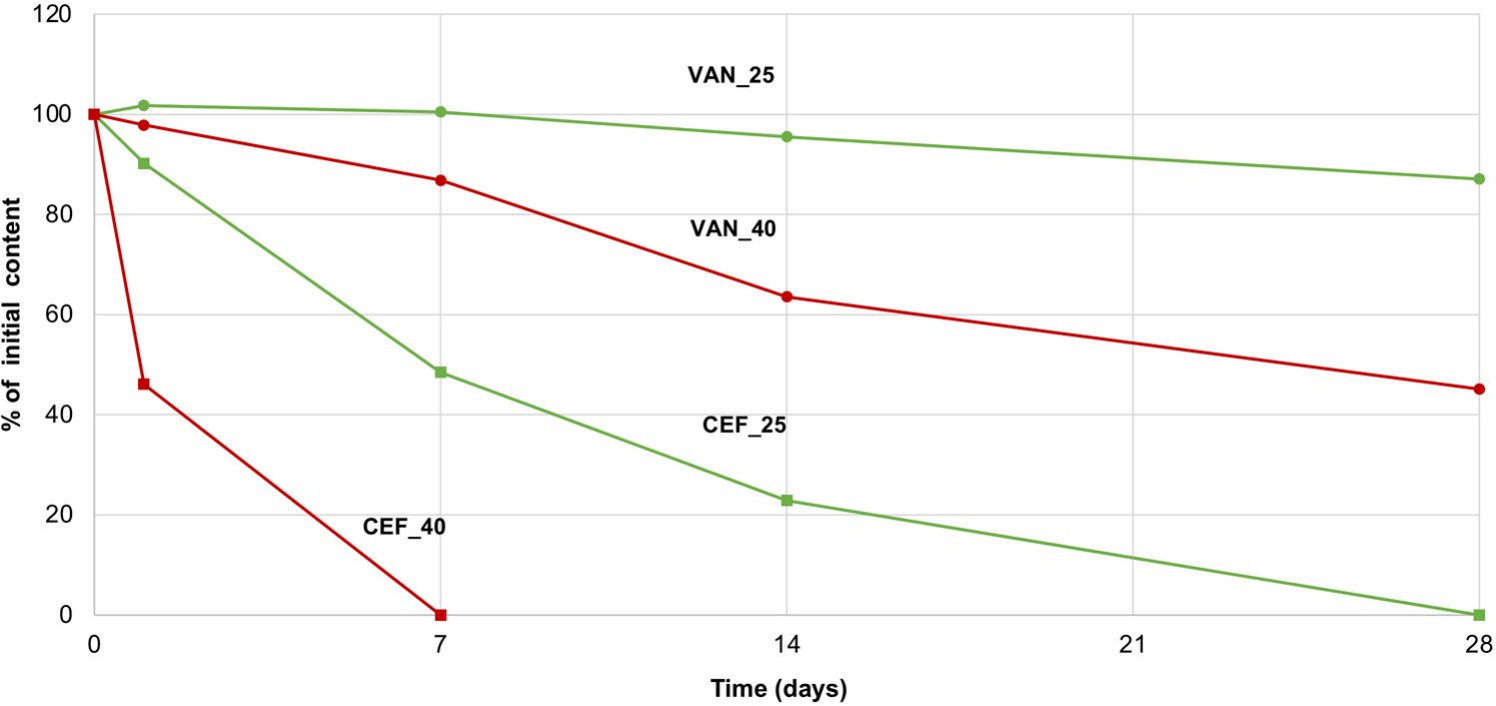
Stability of CEF and VAN in 5% aqueous solutions stored for 4 weeks at a temperature of 25 °C (VAN_25, CEF_25) and 40 °C (VAN_40, CEF_40).

**Figure 7.**
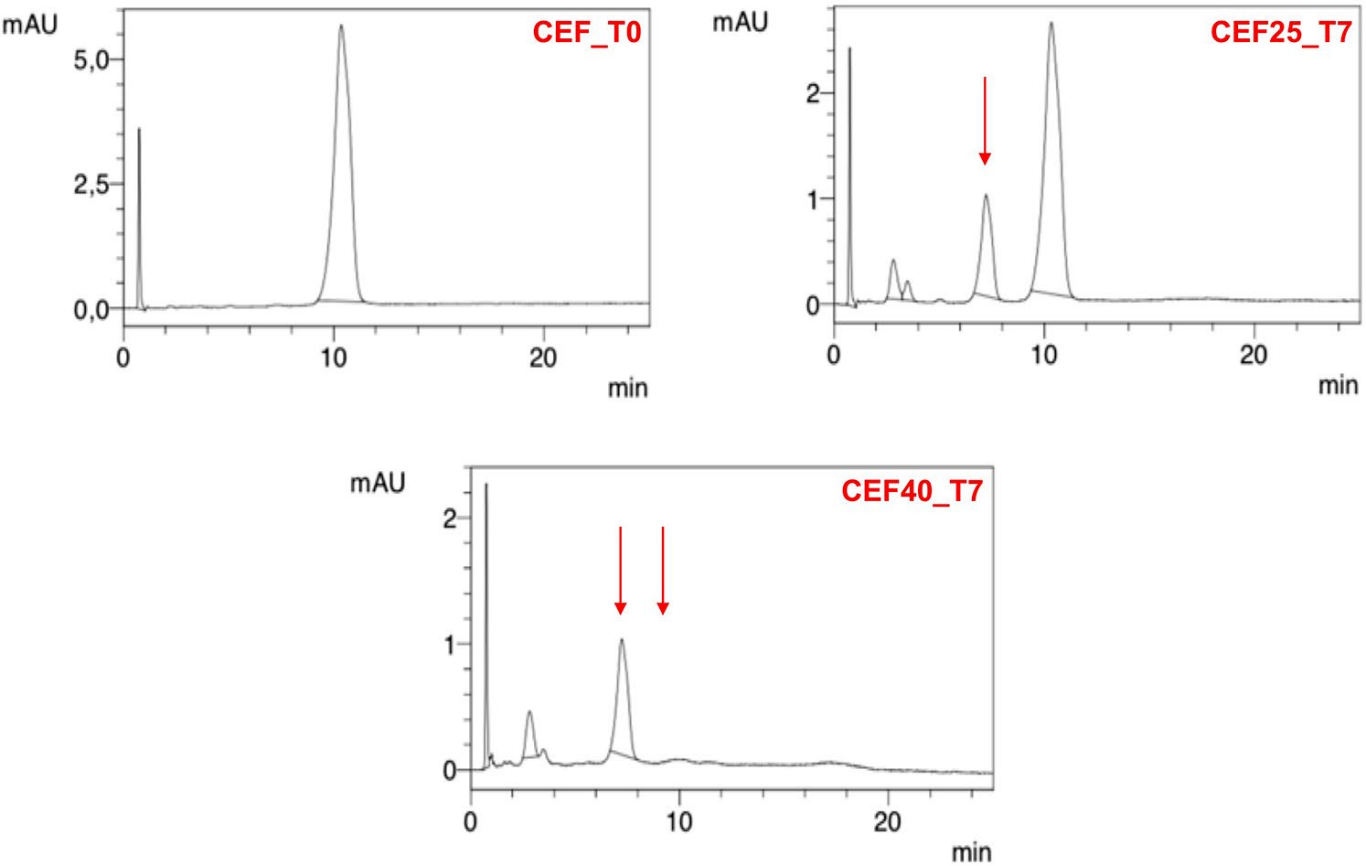
HPLC chromatograms of 5% CEF solution (CEF_T0) in comparison with the solution stored for seven days at 25 °C (CEF25_T7) and 40 °C (CEF40_T7).

For CEF solution, a change in the color from yellow to dark red along with a simultaneous decrease in pH from 9.2 to 8.5 was noticed. Already after 24 hours at 40 °C, a large decrease in the CEF content was observed, and after seven days, HPLC analysis revealed a complete degradation of the antibiotic (Figure 6) with only one main peak indicating the appearance of the degradation product, i.e. descarbamoyl CEF (Figure 7). At 25 °C, degradation occurred more slowly, and it was only after 4 weeks of storage that complete degradation of CEF was observed in the solution.

**Figure 8.**
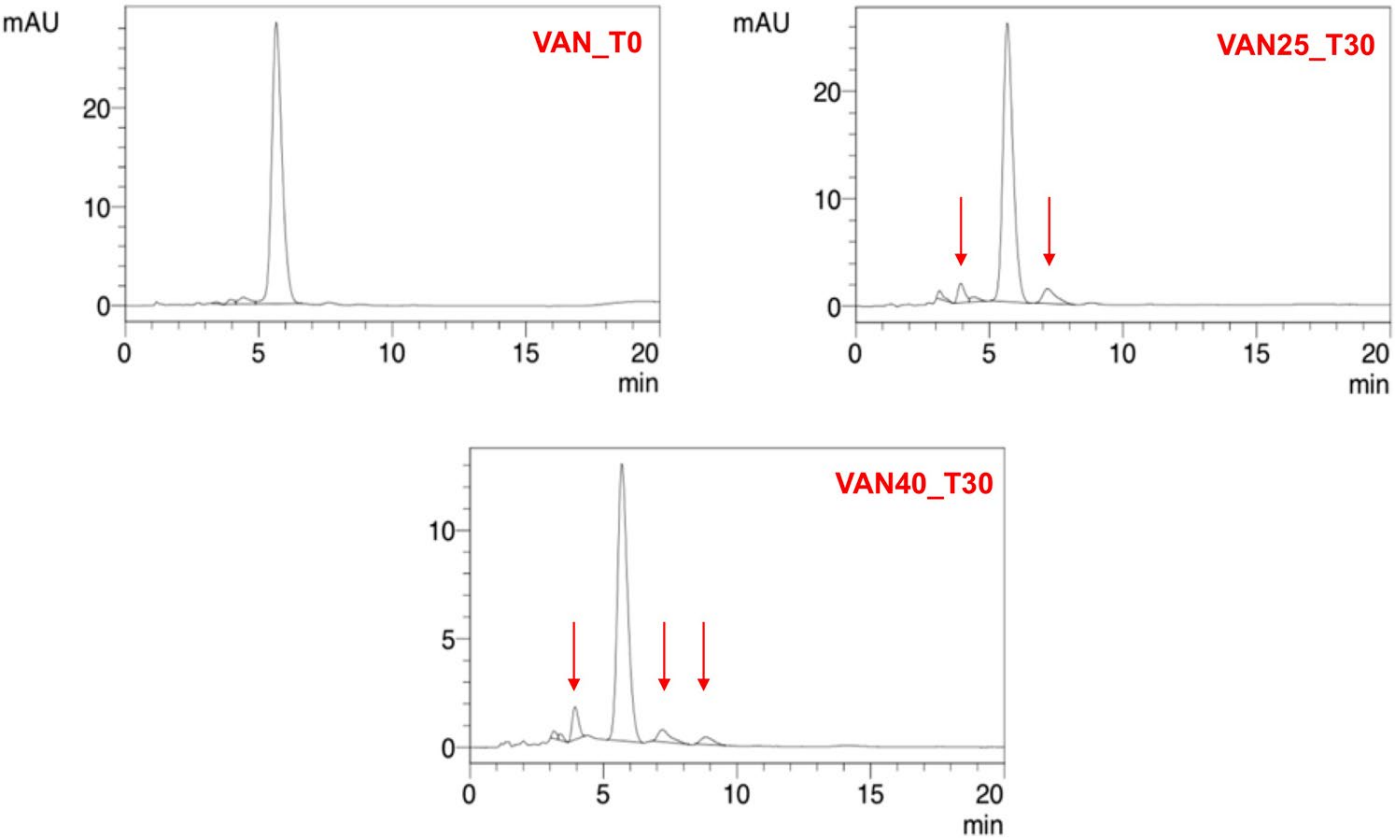
HPLC chromatograms of 5% VAN solution (VAN_T0) in comparison with the solution stored for 30 days at 25 °C (VAN25_T30) and 40 °C (VAN40_T30).

On the other hand, VAN in the aqueous solution demonstrated better stability. After one month of storage at 25 °C, 87% of the initial content was assayed by HPLC, while in the solution stored at 40 °C, it was 45% of the drug non-degraded (Figure 6). The degradation of VAN and the appearance of degradation products could be observed in the solution through the presence of crystalline solids, which were identified in HPLC chromatograms (Figure 8) as the main degradation products of VAN: CDP-I-major and CDP-I-minor (Suchý et al., 2016; Klapkova et al., 2020). The changes in the pH were not noticed.

#### 3.3.2. Stability of CEF and VAN in SEO suspensions

**Figure 9.**
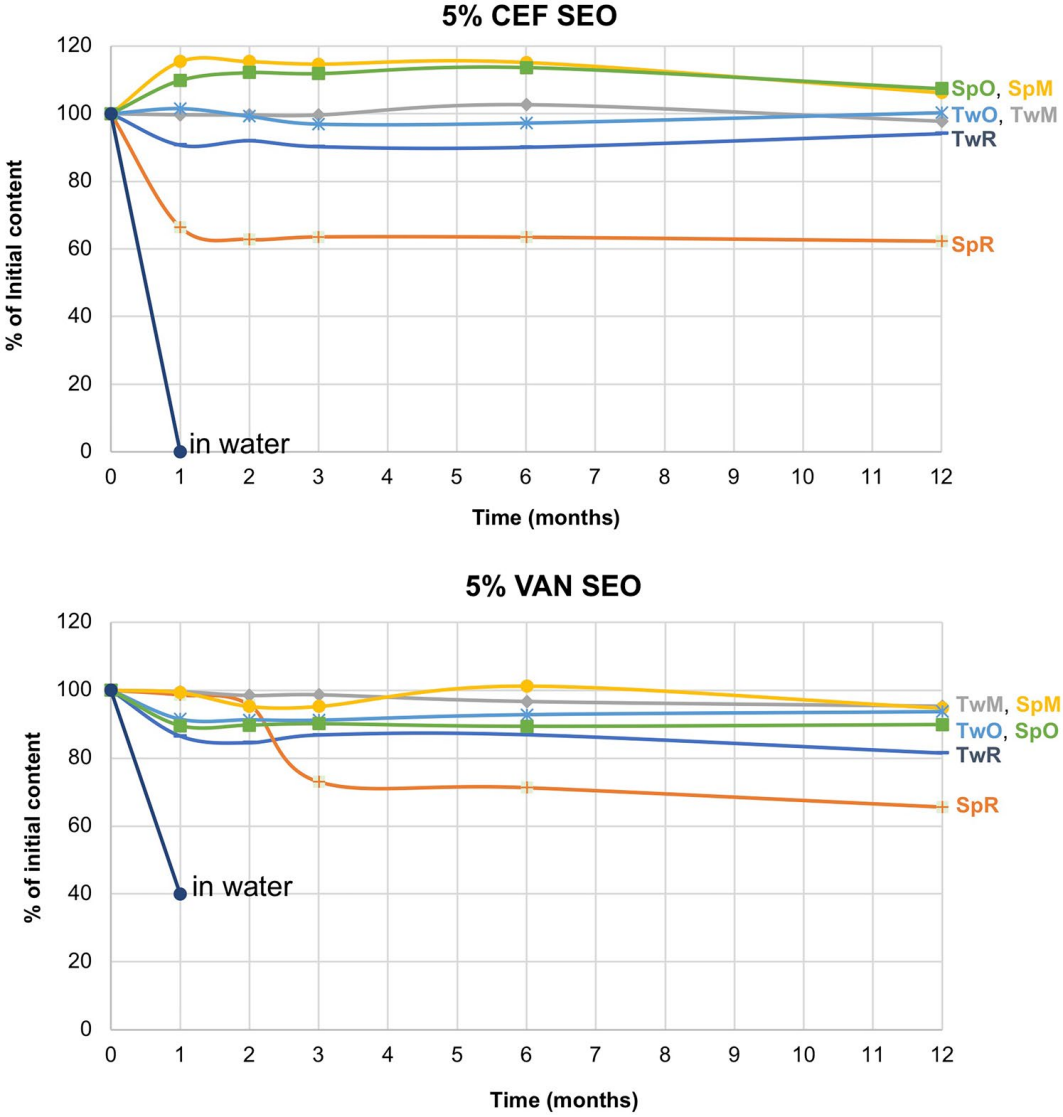
Changes in the content of CEF and VAN in the tested SEO suspensions during one year of storage at 40 °C (the formulation symbols correspond to the compositions presented in Table 1).

Figure 9 depicts the observed changes in the concentration of the investigated antibiotics during their storage in the form of SEO suspension for one year at a temperature of 40 °C.

**Figure 10.**
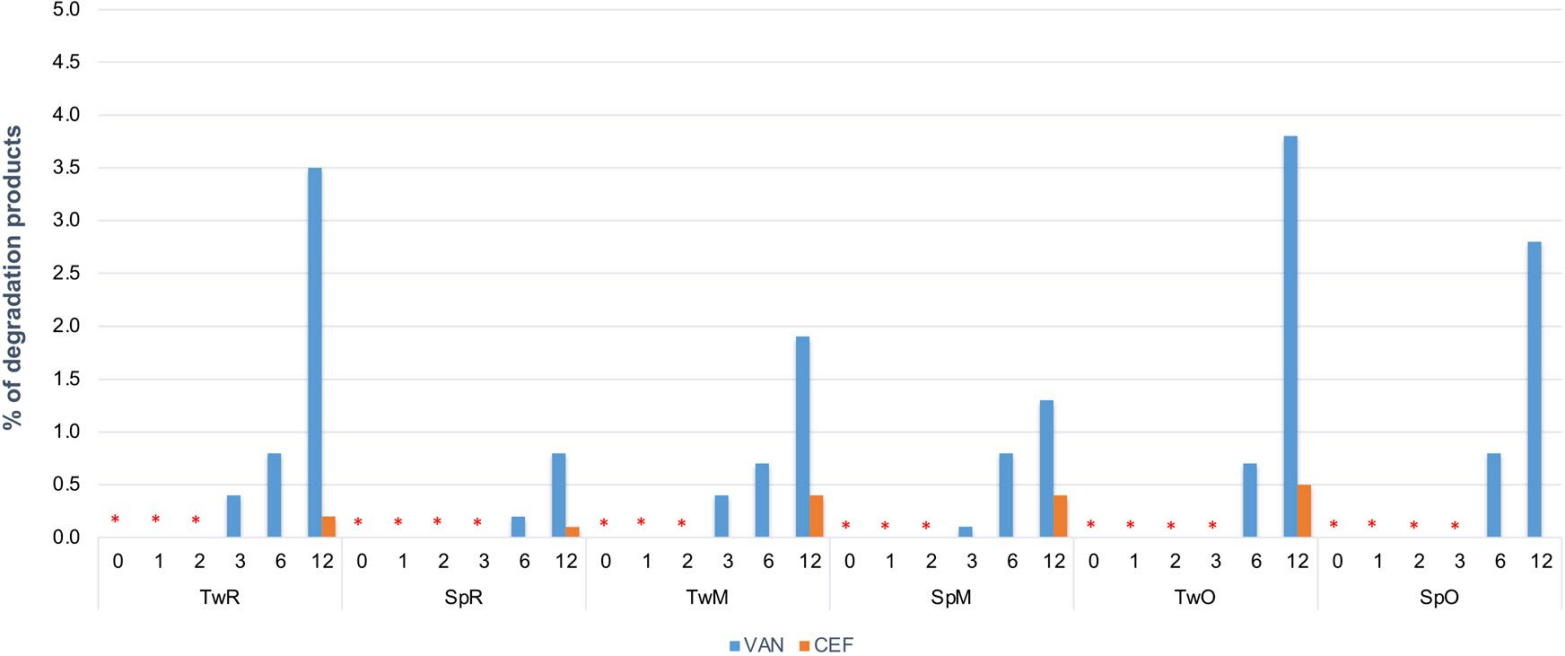
Changes in the content of degradation products in CEF and VAN SEO suspensions during storage at 40 °C for 12 months. *No peaks indicating the presence of degradation products were observed.

Under accelerated storage conditions, CEF remained stable for 1 year in all SEO formulations except for CEF_SpR, where after one month of storage, the CEF concentration was reduced to 64% of the initial content. Importantly, in this formulation, the antibiotic content remained consistent until the end of the study. It must also be noted that the loss of CEF was not compensated by the increased degradation products since the additional peaks were only observed in the chromatogram of CEF_SpR after 12 months of storage at 40 °C, with small quantity of 0.2% (Figure 10). A similar observation was also made for VAN SEO formulations, as described below.

**Figure 11.**
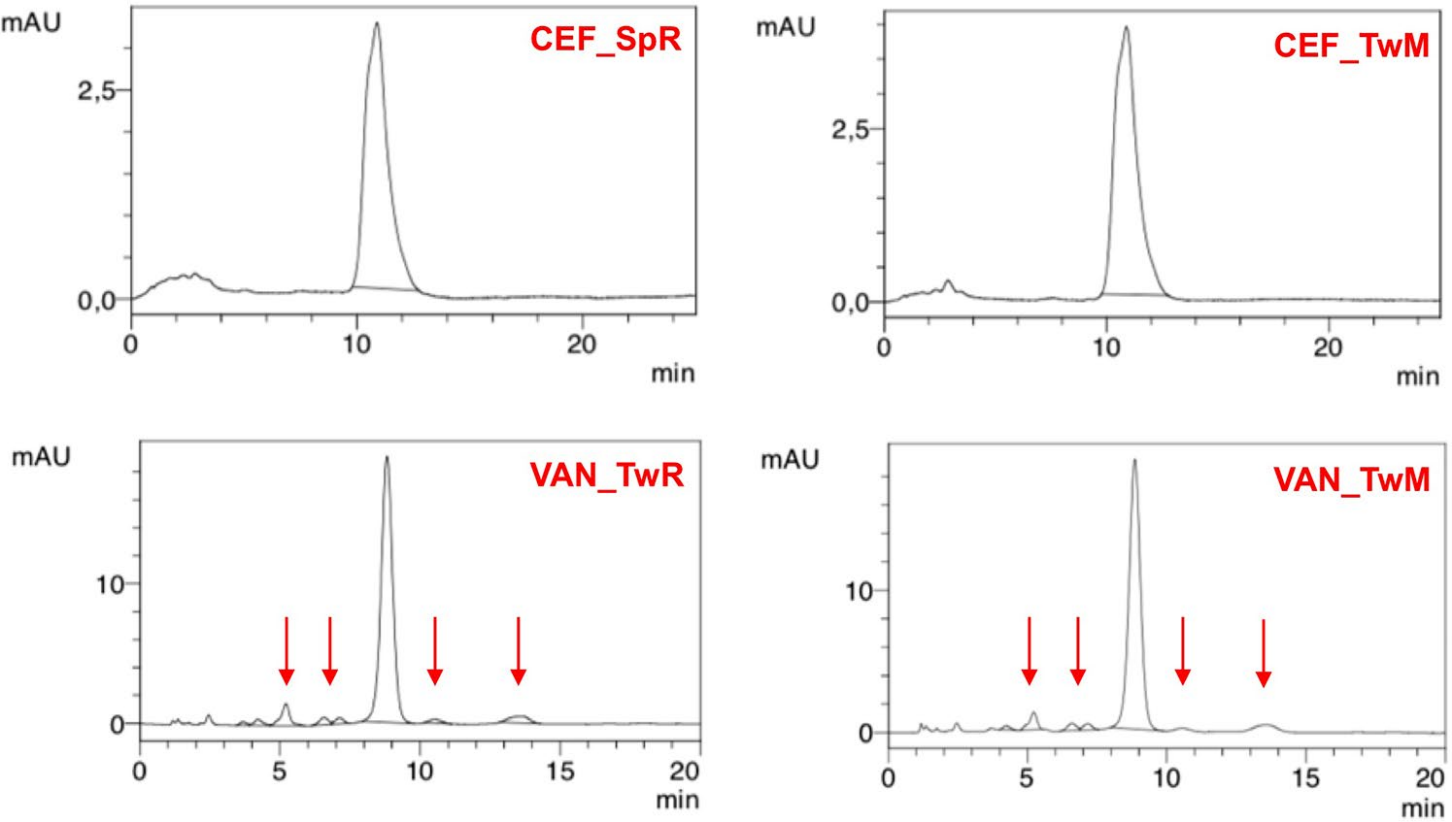
Examples of HPLC chromatograms of SEO suspensions stored for a year at 40 °C.

Comparable to CEF, regardless of the type of surfactant used, VAN was stable in all formulations, except the one with castor oil (VAN_SpR, VAN_TwR). The content of antibiotics in SEO containing Miglyol or olive oil, after one year at 40 °C was not lower than 90% of the initial content (Figure 9). The content of degradation products in formulations with Tween 80 was only slightly higher than with Span. In none of the formulations stored at 40 °C did the content of degradation products exceed 5% (Figures 10 and 11).

The reason for the low measured VAN and CEF content in formulations with castor oil and Span (Figure 9), despite the absence of peaks indicating the presence of degradation products, may be attributed to its high density, which poses particular challenges in redispersing the antibiotic sediment. After 12 months of storage, the suspensions with castor oil required at least one minute of vortex mixing, otherwise, the suspension remained non-uniform.

Although not so evident, the problem with redispersion of the sediment appeared in all other SEO formulations with VAN or CEF, stored for 1 year at 40 °C. However, generally 30 seconds of vortex mixing were sufficient to obtain homogenous suspension.

All SEO suspensions subjected to the stability test emulsified easily with water and no differences were observed in the appearance, droplet size, pH, or zeta potential of the resulting emulsion (Table 6).

**Table 6.** Characteristics of emulsions prepared from the stored SEO suspensions (12 months at 40 °C).

Name	Oil	Surfactant	Time	Zeta potential (mV)	pH	*d*_50_ (µm)
*CEF*						
CEF_TwM	Miglyol	Tween 20	T0	−41.7 ± 0.7	6.4	6.2 ± 0.3
			12 months	−42.3 ± 0.5	6.4	6.0 ± 0.3
CEF_SpM		Span 80	T0	−68.4 ± 1.0	6.4	7.9 ± 0.6
			12 months	−65.4 ± 0.8	6.4	8.1 ± 0.8
*VAN*						
VAN_TwM	Miglyol	Tween 20	T0	−31.9 ± 0.6	5.7	5.7 ± 0.3
			12 months	−34.3 ± 0.3	5.8	5.2 ± 0.2
VAN_SpM		Span 80	T0	−40.7 ± 0.7	5.8	4.1 ± 0.4
			T_12_	−41.9 ± 0.8	5.7	4.5 ± 0.5

#### 3.3.3. SEO suspensions with citrate sodium

To facilitate redispersion of the antibiotic sediment in the tested SEO suspensions, the formulations containing 2% (w/w) sodium citrate were prepared. The effect of this anionic modifier was investigated in formulations containing Miglyol, selected as the most promising for further studies. Miglyol is colorless and has a refractive index of 1.45 which is similar to the index of the aqueous layer of natural tears (1.35) (Ammar et al., 2009). It is considered that the refractive index of the ophthalmic formulation should closely match that of tear fluid. A significant disparity in these values increases the likelihood of causing visual disturbances, which can result in poor patient compliance and treatment failure.

Citrates are used in suspensions to provide surface charge on particles, preventing agglomeration. In this regard, phosphates can also be used. However, there is increasing scientific evidence suggesting that phosphate ions, when in contact with calcium ions present in the rod cells of the eye, can lead to the formation of deposits, ultimately resulting in cloudiness of vitreous body. Therefore, there is currently a caution against adding them to eye preparations. Manufacturers of products that previously contained phosphates are transitioning to citrates. An example of a product where phosphate buffer was replaced with citrate is Hylo-Comod (Ursapharm, Saarbrücken, Germany), used as ‘artificial tears’ for dry eye conditions (Bernauer et al., 2006).

Accelerated aging tests were conducted by storing the CEF_TwMc, VAN_TwMc formulations for 6 months at 40 °C.

It was found that the addition of sodium citrate made it very easy to redisperse the sediment even after 6 months of storage. Manual shaking of the vial was sufficient, and there was no need for vortex mixing which was required in the previous studies. Upon adding water to the SEO suspension, both the citrate and the antibiotic dissolved quickly, resulting in the formation of a homogeneous emulsion.

**Figure 12.**
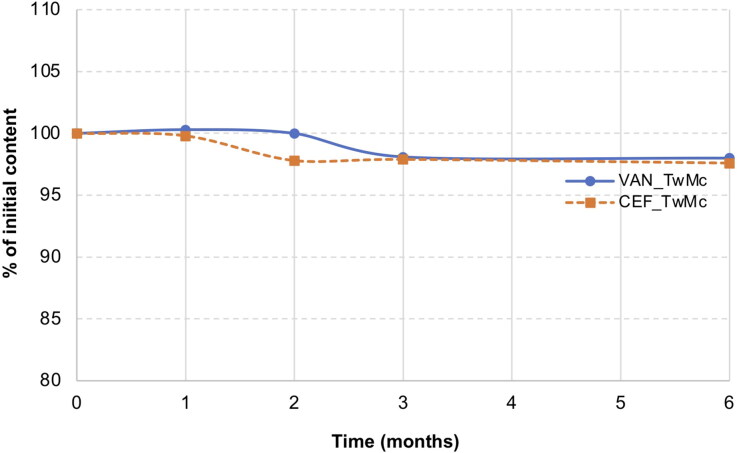
Stability of CEF and VAN in SEO (Miglyol and tween 20) with citrate sodium stored for 6 months at 40 °C.

Following the HPLC analysis of the samples stored for 6 months at 40 °C, no peaks indicating CEF degradation were observed. In the case of VAN, peaks originating from degradation products were observed on the chromatograms after 3 months of storage. However, the content of degradation products did not exceed 0.9% and remained at the same level for up to 6 months (Figure 12).

Table 7 presents the characteristics of emulsion prepared from SEO suspensions with sodium citrate. It was evident that storage of the preparation at 40 °C for 6 months did not affect its emulsification potential.

**Table 7.** Physicochemical parameters of emulsions prepared from the stored (6 months, 40 °C) SEO (Miglyol and tween 20) suspensions with CEF or VAN containing sodium citrate.

Time (months)	pH	Zeta potential (mV)	Median droplet size (*d*_50_) (µm)
*CEF_TwMc*			
0	7.1	−26.6 ± 0.4	5.33 ± 0.4
6	7.1	−26.8 ± 0.2	5.42 ± 0.5
*VAN_TwMc*			
0	6.5	−28.3 ± 0.6	5.92 ± 0.3
6	6.5	−28.6 ± 0.7	6.06 ± 0.2

### 3.4. Microbiological assay

When evaluating the chemical stability of an antibiotic-loaded pharmaceutical formulation, it is important to acknowledge the limitations of the method. Simply relying on chemical analysis may not always provide a complete confirmation of retained potency. Even if the chemical analysis indicates the presence of the active substance, the product can undergo changes that alter its antibacterial effectiveness. Additionally, there is the possibility that newly formed degradation products might exhibit some level of antimicrobial activity. Nevertheless, previous literature (Griffiths et al., 2006) has demonstrated that microbiological potency assessments, such as those for VAN, yield very similar results to the assessments of its chemical stability using HPLC methods. This indicates that the results from both methods progress in a similar manner and show a strong agreement.

To confirm the microbial effectiveness of VAN in SEO carrier and eliminate potential interference from any of the components, a microbiological assay was conducted following a method outlined in the European Pharmacopeia (Ph. Eur. 11) with slight adjustments. As specified in the Ph. Eur. (chapter 2.7.2), the potency of an antibiotic is determined by comparing the inhibition of the growth of sensitive microorganisms caused by known concentrations of the antibiotic in a reference formulation.

**Figure 13.**
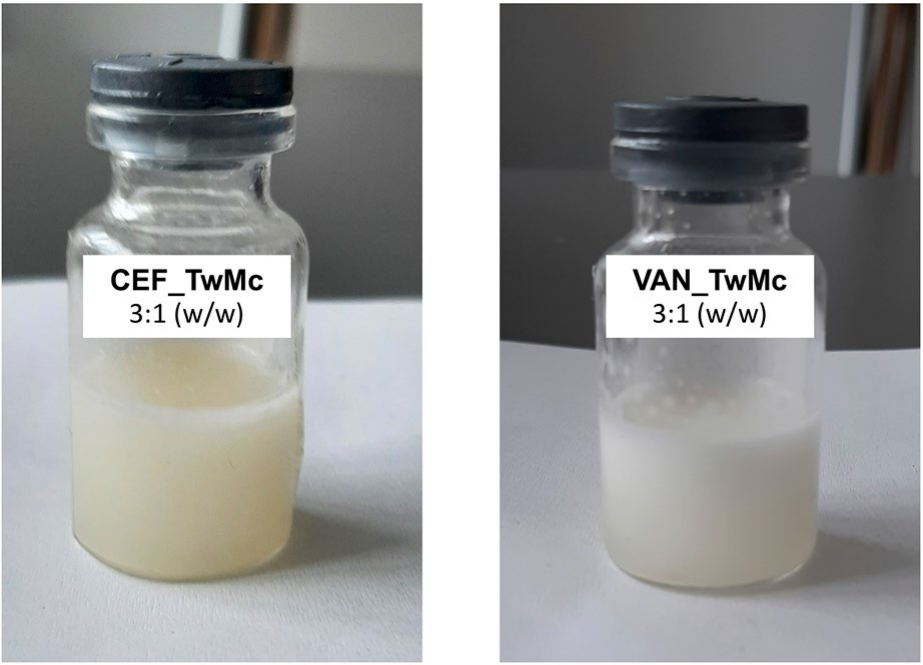
SEO (Tween 20 and Miglyol) emulsions with CEF or VAN, with the addition of sodium citrate, obtained by diluting the suspension with water at a ratio of 3:1 (w/w).

In the performed study, SEO with Tween and Miglyol, containing CEF or VAN, both immediately after preparation and after one year of storage at 25 °C were analyzed. The suspensions were diluted with water at a 3:1 (w/w) ratio (Figure 13). This dilution was chosen to account for the fact that the volume of tear fluid in the conjunctival sac is approximately 7 µl (Rentka et al., 2017), while the volume of a single eye drop is around 20 µl. Administering one drop of the preparation into the conjunctival sac, following the concept of the presented systems, results in a diluted emulsion with a 3:1 ratio, containing the antibiotic in the initial concentration of 3.75%.

**Figure 14.**
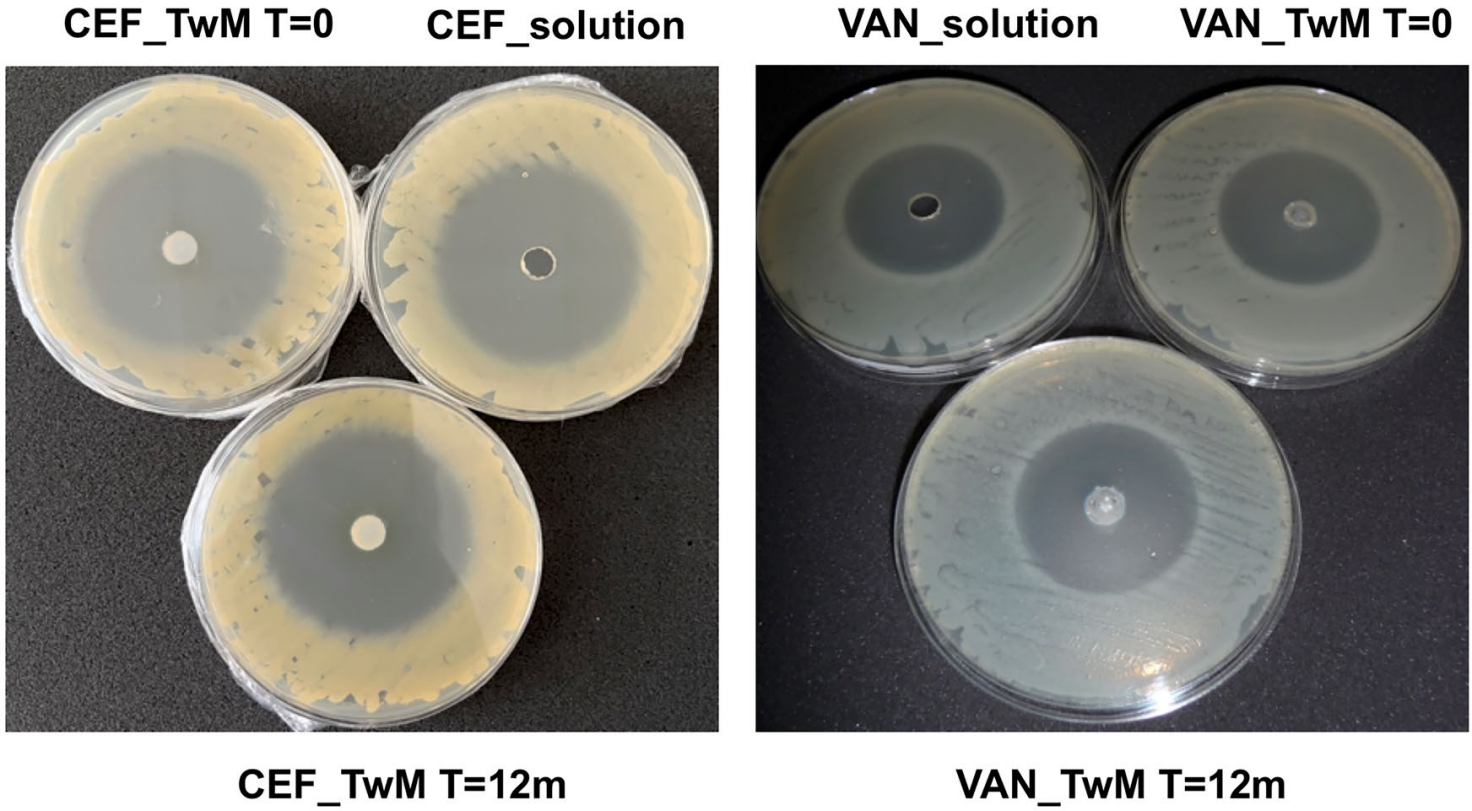
The inhibition zones of tested formulations: with CEF (on the left) and VAN (on the right). Freshly prepared SEO formulations and antibiotic solutions (*T* = 0) and SEO formulations after 12 months of storage at 25 °C (*T* = 12 months) are compared.

Figure 14 illustrates the inhibition zones obtained in the microbiological study of SEO formulations containing Miglyol and Tween 20 with CEF or VAN, stored for 12 months at 25 °C. This is compared to freshly prepared SEO formulations with the same composition and freshly made solutions of these antibiotics. Freshly prepared SEO formulations with the same composition and solutions of these antibiotics used as controls were also assessed. Photographs and measurements were taken after 24 hours incubation at 37 °C.

The size of the growth inhibition zone for *S. aureus* on the plates where CEF was applied was approximately 52 mm, while on the plates with VAN formulations the growth inhibition zone for *B. subtilis* was around 41 mm (Table 8).

**Table 8.** The inhibition zones (mean ± SD) measured in the microbiological study of the freshly prepared (*T* = 0) and stored formulations (*n* = 5).

Formulation – antibiotic concentration 3.75% (w/w)	Inhibition zones (mm)	
	CEF	VAN
Aqueous solution	51.6 ± 0.5	41.4 ± 0.5
Emulsion from SEO_TwM (*T* = 0)	51.8 ± 0.5	41.4 ± 0.4
Emulsion from SEO_TwM (*T* = 12 months)	51.8 ± 0.2	41.2 ± 0.4

As a result of the conducted research, it was observed that the size of inhibition zones for CEF and VAN SEO-emulsions was the same as the size of inhibition zones for the reference solutions. It was concluded that both for CEF and VAN, the SEO carrier did not reduce microbiological activity of these antibiotics. It was concluded that the SEO carrier did not reduce microbiological activity both of CEF and VAN.

Furthermore, no statistical differences were noted in the size of inhibition zones on the plates where emulsions obtained by diluting freshly prepared SEO with antibiotics were applied, compared to those that were stored for a year at 25 °C.

## 4. Conclusions

The concept of the research aimed to develop a novel carrier suitable for ocular delivery of antibiotics that are unstable in an aqueous environment. This is the first study reporting the potential of SEO as a universal and simple, yet safe and effective, carrier for ocular administration of water-soluble antibiotics, forming immediately after administration *in vivo* an emulsion, with fast dissolution of antibiotic.

Our findings proved very good chemical stability of CEF and VAN in all tested SEO formulations except for the formulations with castor oil, which exhibited poor emulsification and an unexplained decrease in the drug content.

During storage, slight problems with redispersion of the sediment in suspensions occurred but the addition of sodium citrate in concentration of 2% (w/w) was found to be sufficient for easily obtaining a homogeneous suspension, solely through manual shaking. In accelerated aging tests, it was also demonstrated that sodium citrate does not affect the stability of the tested antibiotics.

The activity of antibiotics in the prepared SEO suspensions was confirmed in microbiological studies when inhibition zones in an agar inoculated with bacteria were compared.

The obtained results are highly optimistic and serve as a basis for further research to assess the eye tolerance of the selected SEO preparations. In the absence of irritant effects, it will be possible to confirm the *in vivo* effectiveness of antibiotics when they are administered as new formulation in clinical trials. Our research focuses primarily on eye drops with antibiotics, but SEO can be a carrier for any other water-sensitive drugs, also administered by other routes, provided it is in contact with the aqueous environment *in vivo* (orally, on mucous membranes, parenterally). Important is that this formulation can be produced on industrial scale, with no limitation of stability or drug concentration.

## Data Availability

Upon reasonable request, data can be obtained from the corresponding author.
